# Premature Coronary Artery Disease due to Homozygous Familial Hypercholesterolemia in a 12-Year-Old Girl

**DOI:** 10.4274/balkanmedj.2017.0490

**Published:** 2018-03-15

**Authors:** Filiz Ekici, Salih Özçobanoğlu, Fırat Kardelen

**Affiliations:** 1 Department of Pediatric Cardiology, Akdeniz University School of Medicine, Antalya, Turkey; 2 Department of Cardiovascular Surgery, Akdeniz University School of Medicine, Antalya, Turkey; 3 Department of Pediatric Cardiology, Akdeniz University School of Medicine, Antalya, Turkey

**Keywords:** Premature coronary artery disease, homozygous familial hypercholesterolemia, children, coronary surgery

## Abstract

**Background:**

Homozygous familial hypercholesterolemia is a rare inherited metabolic disease caused by low-density lipoprotein receptor abnormality. Patients with homozygous familial hypercholesterolemia have an increased risk of cardiovascular complication that usually occurs in the first decade of life. Here, we report a 12-year-old girl with an unpredicted presentation for coronary artery disease and found to have homozygous familial hypercholesterolemia.

**Case Report:**

A 12-year-old girl was admitted to our unit with syncope. Chest X-ray showed bilateral diffuse pneumonic consolidation and mild cardiomegaly. We detected stable ST depression by electrocardiography. Echocardiography showed normal systolic functions. Troponin-1 levels were high (66 mcg/dL, upper limit: 0.04 mcg/dL). Influenza A virus DNA was detected by the respiratory viral panel. After her successful treatment for acute pneumonia and myocarditis due to Influenza A virus, her syncope attacks persisted. Marked ST elevation was observed during exercise electrocardiography. Coronary angiography showed severe occlusions in the coronary arteries. High serum levels of total cholesterol (756 mg/dL) and low-density lipoprotein-C (556 mg/dL) were noticed. She had no tendon xanthomas. Medical histories revealed that her family members were diagnosed with heterozygous familial hypercholesterolemia. A coronary bypass surgery was performed. Statin and ezetimibe treatments were started. We also planned lipid apheresis.

**Conclusion:**

Children with homozygous familial hypercholesterolemia may present with symptoms of premature coronary heart disease requiring a routine lipid test and careful anamnesis.

Homozygous familial hypercholesterolemia (HoFH) is a hereditary, metabolic disease caused by low-density lipoprotein (LDL) receptor abnormality (1). HoFH is a rare disease with an estimated prevalence of 1 per 300.000 in European populations (1,2). Patients with HoFH have an increased risk for cardiovascular disease (CVD) that usually occurs in the first decade of life. Myocardial infarction and death can be seen before 20-years-old (1,2,3,4). Here we report a 12-years-old girl with an unpredicted presentation for coronary artery disease (CAD) related to HoFH.

## CASE PRESENTATION

A 12-year-old girl was admitted to our unit with syncope. She had fainted after climbing the stairs. She was born to non-consanguineous parents. Her history was negative for systemic disease, and she denied taking any medicine or exposure to toxins. She had experienced two syncope attacks during exercise, four months previously.

**Figure 1:**
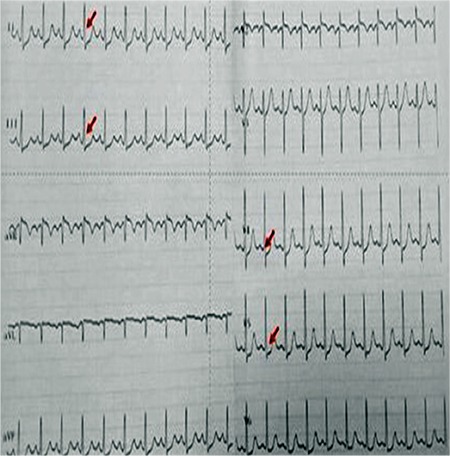
Upon admission, electrocardiography showed an ST depression in the inferior leads and lateral precordial derivations.

**Figure 2:**
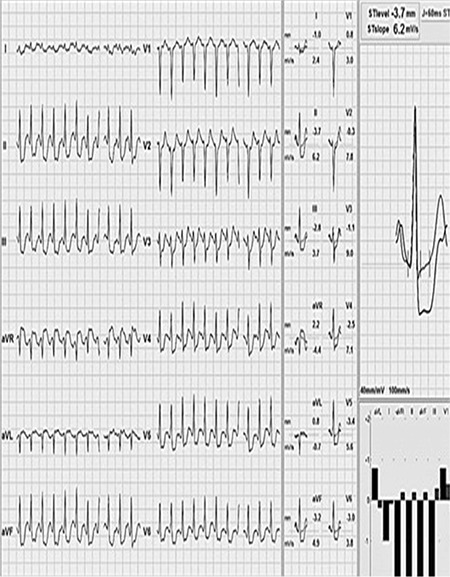
A cardiovascular treadmill stress test showed significant ST depression in the inferior leads and lateral precordial derivation.

**Figure 3:**
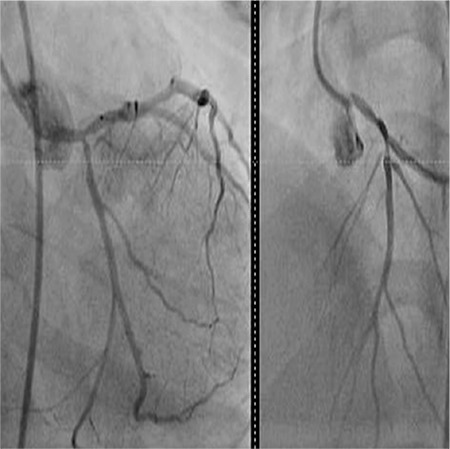
Conventional coronary angiography revealed a significant occlusion of the left anterior descending artery and circumflex artery.

By physical examination, her the heart rate was 95 beats min-1, respiratory rate was 25 min-1, and blood pressure was 90/55 mmHg. Body temperature was 37 °C. While she was awake, the respiratory sounds were diminished. Laboratory examinations showed leukocytosis (13.5000 mm^3^) and elevated acute phase reactants (erythrocyte sedimentation rate: 83 mm/h; C-reactive protein: 8.2 mg/dL). Chest X-ray showed bilateral diffuse pneumonic consolidation and mild cardiomegaly. Except for a stable ST depression, electrocardiography (ECG) was normal (Figure 1). Echocardiography showed thickening of the mitral and aortic valves and mild mitral valve insufficiency. The left ventricle was mildly dilated, but systolic function was within normal limits (the ejection fraction was 68%). The troponin-1 level was high (66 mcg/dL, upper limit: 0.04 mcg/dL). Influenza A virus DNA was detected by respiratory viral panel screening. The toxicologic screening was negative. Throat, blood, and urine cultures were negative. Antistreptolysin O titer was within normal limits. Cranial computed tomography and electroencephalography were normal. We did not detect any arrhythmia by 24 h ambulatory electrocardiographic monitoring. Intravenous immunoglobulin was given for treatment of myocarditis. We started combined wide-spectrum antibiotics and antiviral oseltamire treatments. At the third day, she had no complaint; acute phase reactants and cardiac markers were negative. Normal cardiac size and clear lung fields were detected by chest X-ray. We stopped giving antibiotics after ruling out bacterial infection. However, there was a stable ST depression in the inferior leads by ECG. Coronary angiography by computed tomography displayed a suspected atheroma plague in the left anterior descending (LAD) aorta. Meanwhile, she had experienced another syncope attack. ECG showed an ST depression in the inferior leads, but the cardiac marker was negative. A cardiovascular stress test showed significant ST depression in the inferior leads (Figure 2). Conventional coronary angiography revealed a significant occlusion of the LAD artery and circumflex artery (Figure 3). High serum levels of total cholesterol (756 mg/dL) and LD-cholesterol (LDL-C) (556 mg/dL) were noticed. Carotid artery Doppler imaging showed moderate occlusion in both carotid arteries. When the patient’s medical history was re-taken, we found that her grandmother had been diagnosed with heterozygous familial hypercholesterolemia (HeFH) and underwent a coronary bypass surgery at 50-years-of-age. Her parents and 4-year-old sister’s blood analyses showed hypercholesterolemia, which was suggesting of HeFH. Diagnosis of HoFH was made in our case based on elevated plasma cholesterol levels, premature CAD, and positive family history. Her coagulation and thrombus panel and thyroid function tests were normal, and metabolic screening tests were negative. She had no tendon xanthomas. A coronary bypass surgery was performed between the left internal mammary arteries (LIMA) and LAD. Rosuvastatin, ezetimibe (a calcium-canal blocker drug), and aspirin treatments were started. After 1 year, she was in good condition and had no complaint. Her electrocardiography was normal. On echocardiogram, mitral and aortic regurgitations persisted. The LDL cholesterol level was slightly decreased (350-450 mg/dL), and we have planned lipid apheresis. She was consulted by the Pediatric Gastroenterology and Transplantation committee at our hospital. Meanwhile, the combined and high doses of ezetimibe and rosuvastatin therapy have resulted in a 37% reduction in her serum LDL level. She has remained free from treatment-related adverse responses, and CVD during follow-up and her parents did not accept liver transplantation. Written informed consent was obtained from the parents.

## DISCUSSION

Here we presented a child with premature CAD related to HoFH. Clinical and laboratory findings resembled subclinical myocarditis and, coincidentally, she had pneumonitis due to Influenza A virus. After a detailed assessment, she was found to have CAD due to HoFH.

Pediatric familial hypercholesterolemia (FH), is usually diagnosed phenotypically by the presence of an LDL level consistent with FH, a family history of premature coronary heart disease, high baseline cholesterol in one parent, an FH-causing mutation, or a combination of these (1). Cholesterol testing should be used to make a phenotypic diagnosis, and secondary causes of hypercholesterolemia should be ruled out. Children with dyslipidemia usually have no symptoms; they are usually diagnosed by tendon xanthomas, which is a pathognomonic finding for HoFH and seen in 20-40% of cases. Multiple xanthomas (nodules) are usually noticed on the skin, Achilles tendons, and fingers. Earlier symptoms are typically related to aortic stenosis and regurgitation due to massive accumulation of cholesterol in the aortic valve or coronary ostium, and syncope may result from these lesions (1). The first cardiovascular events in patients with HoFH can develop in early childhood but usually occur during adolescence, depending on the severity of the mutations (3,4,5,6,7). The diagnosis of our case and her parents were made based on plasma LDL-C levels and history of CAD in her family members. The pedigree of our case suggested that she had heterozygous parents, a grandmother and a brother that each might carry one copy of an FH-mutation-bearing allele, so our case must have two copies of FH-mutation-bearing alleles. Up to 40% of the children (2,6) and 59% of adult patients (7) with HoFH suffer from a cardiovascular event. Our case had thickening and regurgitation of aortic and mitral valves, but there was no stenosis in the aortic valve or coronary ostium. The chief complaint of our case was repeated syncope that might be related to moderate occlusion in the carotid arteries, which was demonstrated by carotid artery Doppler imaging.

The treatment options are reduced fat diet, drugs, lipid apheresis, and liver transplantation. Statins are safe and effective at lowering LDL in children, can restore endothelial function and regress the thickening of the intima of the vessel wall (5), and have been shown to reduce cardiovascular and all-cause mortality. The expert consensus report recommends a target LDL level of 130 mg/dL from 10-years-of-age, or ideally a 50% reduction from pre-treatment levels for children between 8- and 10-years-of-age (1). However, only mild reduction (10-25%) in the LDL plasma levels is reported in most patients with HoFH, even at the highest doses of the most efficacious statins (1,5). Combinations with bile-acid sequestrates, niacin, fibrates, and probucol have been used successfully. An early combination therapy with LDL apheresis, statins, and cholesterol absorption inhibitors in children with high-risk conditions or other major risk factor are recommended by the consensus report (1,5,6). Although there are no data available for children, oral lomitapide and injectable mipomersen have recently been approved in the United States (US) as adjunct therapies for HoFH in patients aged ≥18 and ≥12 years, respectively (1). 

Our case has possible (not definite) HoFH; since there is significant phenotypic overlap between HeFH and HoFH cases, a genetic diagnosis is preferred whenever possible. Genetic diagnosis can also guide therapy. For example, PCSK9 inhibitors are not effective in HoFH patients with no residual LDRL activity, whereas they may prove effective in double heterozygous FH cases. 

LT might be performed in medically resistant severe cases (1,6). Early diagnosis is crucial to prevent premature CVD and for providing long-term survival in cases with HoFH (1,2,3,4,5,6). Lipid apheresis is recommended at a very early age (<6-7 years) for the prevention of CAD in homozygous patients (7). Since HoFH is a hereditary metabolic disorder, liver transplantation is the only curative treatment and preemptive liver transplantation or lipid apheresis before the onset of CVD might provide significantly better outcome (6,7). However, combined and intensive therapy is the first choice of treatment in many cases due to unavailability of apheresis for liver transplantation. Lin et al. (8) have observed a greater than 40% reduction in LDL cholesterol and no drug-related adverse responses and cardiovascular events in four Chinese children following a triple combination of atorvastatin, probucol, and ezetimibe during at least a 6-year follow-up period. As implicated by Lin et al. (8), we might give probucol to our case according to her disease progression and her lipid profile.

Coronary revascularization therapy might be required when symptoms or ischemia develop due to CAD (1,9,10,11). The treatment of the coronary stenosis with coronary artery bypass grafting (CABG) and percutaneous coronary intervention (PCI) has been described previously (9,10,11). Based on an individual assessment of the cases, either option can be preferred. The coronary vascular structures and patient age should be assessed carefully. There are no available studies including children with HoFH and comparing the vascular interventional options. Case series implicate that CABG is the first choice in cases with severe form CAD and is performed in almost 10% of cases with CAD (9,10,11). Alim et al. (6) have performed LT for HoFH in eight children with a median age of 10 years. CAD was seen in three of their patients (38%) and CABG was performed in one patient. Coronary revascularization in childhood is a rare procedure; the youngest patient with HoFH undergoing CABG was reported by Oral et al. (12). They performed CABG and liver transplantation in a 6-year-old boy. Bilal et al. (10) described a 12-year-old child with HoFH that underwent triple coronary bypass. In a recently published paper, Nazif et al. (13) presented a 3-year-old boy with HoFH and severe CAD, and the case was managed with PCI with bioresorbable scaffolds for the first time. CABG between LIMA and LAD was established in our case because of the involvement of multiple vessels and ischemic symptoms. However, surgery could not be performed in right coronary artery, because of technical difficulties related to the small distal arterial targets, and potentially decreased graft patency that was predicted during surgery. However, to the best of our knowledge, our case is one of the youngest cases with HoFH in the literature for whom coronary revascularization has been done in childhood.

Familial hypercholesterolemia is usually underdiagnosed and untreated in the general population. HoFH should be kept in mind as a cause of myocardial ischemia in children. Early diagnosis can be achieved by a high level of clinical suspicion, careful history taking, and population-based screening programs. Similar to adult patients, children with dyslipidemia also require early diagnosis and aggressive treatment strategies.
